# Efficacy and safety of telitacicept in systemic lupus erythematosus with lupus nephritis and nephrotic syndrome: a 12-month retrospective cohort study

**DOI:** 10.3389/fphar.2025.1613790

**Published:** 2025-07-18

**Authors:** Min-Ying Liu, Liu-Jun Li, Ting Li, Hao-Sen Zhu, Chang-Song Lin, Qiang Xu, Qing-Ping Liu

**Affiliations:** ^1^ Department of Rheumatology, The First Affiliated Hospital of Guangzhou University of Chinese Medicine, Guangzhou, China; ^2^ Guangdong Clinical Research Academy of Chinese Medicine, Guangzhou, China; ^3^ The First Clinical Medical School of Guangzhou University of Chinese Medicine, Guangzhou, China; ^4^ Department of Rheumatology, Foshan Hospital of Traditional Chinese Medicine, Foshan, China; ^5^ Department of Nephrology and Rheumatology, Wuyi Hospital of Traditional Chinese Medicine, Jiangmen, China; ^6^ Department of Nephrology and Rheumatology, Traditional Chinese Medicine Hospital Affiliated to Jinan University, Jiangmen, China

**Keywords:** telitacicept, lupus nephritis, nephrotic syndrome, efficacy, safety

## Abstract

**Background:**

This retrospective cohort study evaluated the therapeutic efficacy and safety profile of telitacicept, a novel dual B-cell-activating factor (BAFF)/a proliferation-inducing ligand (April) inhibitor, in managing systemic lupus erythematosus (SLE) patients with lupus nephritis (LN) and nephrotic syndrome (NS), with particular focus on renal and hematological parameters.

**Methods:**

12 SLE patients with biopsy-confirmed LN and NS who received weekly subcutaneous telitacicept (80/160 mg) combined with standard therapies for ≥12 months were analyzed. Primary endpoints include changes in Systemic Lupus Erythematosus Disease Activity Index (SLEDAI) scores, 24-h urinary protein excretion (24hUpr), complement levels (Complement Component 3/Complement Component 4), anti-double-stranded DNA antibodies (anti-dsDNA) titers, immunoglobulin profiles, serum creatinine, and hemoglobin (HGB) at baseline, 3-month, and 12-month intervals. Statistical analysis was performed using SPSS 26.0 and R 4.1.2. The significance level was assessed using a one-sample t-test of the log ratios, with the null hypothesis assuming no effect.

**Results:**

Significant improvements were observed in the cohort (91.7% female, median age 30): SLEDAI: Median reduction from 13 to 4 (p = 0.0029), 24hUpr: 4.0 g/24 h → 0.83 g/24 h (p < 0.001), anti-dsDNA: 120 IU/mL → 13 IU/mL (p = 0.003), Complement restoration: C3 0.56→0.84 g/L; C4 0.1→0.22 g/L (both p < 0.001), HGB improvement: 110→120 g/L (p = 0.0144). Compared to 80 mg dose subgroup, the 160 mg dose subgroup (83.3%) showed superior outcomes with no severe adverse events.

**Conclusion:**

Telitacicept demonstrates robust clinical efficacy in LN-NS management through dual B-cell regulation and complement restoration mechanisms. These practical findings support its potential as a targeted therapy for renal and hematological manifestations of SLE, requiring further validation through randomized controlled trials.

## Key points

• First practical evidence of telitacicept’s renal protective effects in LN-NS comorbidity with higher levels of proteinuria.
• Significant proteinuria reduction (67.5% median decrease at 12 months).
• Dual mechanism of action: B-cell suppression (CD19^+^ reduction 42%) and complement restoration.
• Favorable hematological improvement independent of erythropoietin use.
• Comparable safety profile to belimumab in SLE treatment.

## Introduction

SLE, a multisystem autoimmune disease with complex genetic-environmental interactions (Basta et al., 2020), presents heterogeneous clinical manifestations ranging from cutaneous involvement to life-threatening organ damage. Tian et al. (2023) reported global SLE incidence at 5.14/100,000 person-years (0.4M new cases/year) and prevalence at 43.7/100,000 persons (3.41M affected) (Tian et al., 2023). Among these, LN develops in 40%–60% of SLE patients and represents the strongest predictor of poor long-term outcomes, accounting for 17%–25% of end-stage renal disease (ESRD) cases in young adults (Gasparotto et al., 2020). The co-occurrence of NS in LN patients portends particular clinical challenges, characterized by nephrotic-range proteinuria (>3.5 g/24 h), hypoalbuminemia (<30 g/L), and compensatory hyperlipidemia (Frățilă et al., 2024).

Current therapeutic regimens combining glucocorticoids with immunosuppressants (mycophenolate mofetil, calcineurin inhibitors, cyclophosphamide) achieve complete renal response in only 30%–40% of LN patients within 12 months (Fava and Petri, 2019). Persistent disease activity and treatment-related complications, including opportunistic infections (HR 3.2, 95%CI 1.8–5.7) and cumulative glucocorticoid toxicity, contribute to substantial morbidity (Chen et al., 2023; Parra Sánchez et al., 2022). Although B-cell depletion therapies such as rituximab have shown efficacy in refractory cases (Zucchi et al., 2023), there remains a pressing need for targeted agents with better safety profiles.

The BAFF/April axis has emerged as a linchpin in SLE pathogenesis, with elevated serum BAFF levels correlating with disease activity (r = 0.42, p < 0.001) and renal flares (Arbitman et al., 2022). Telitacicept, a novel recombinant fusion protein combining transmembrane activator and calcium-modulating cyclophilin ligand interactor (TACI) with human IgG-Fc, uniquely acts as a dual inhibitor of BAFF and April (Shi et al., 2021). Following its 2021 approval by China’s National Medical Products Administration (NMPA) for SLE (Dhillon, 2021), more and more evidence suggests therapeutic potential across autoimmune disorders—from IgA nephropathy (24 h proteinuria reduction 48.6% vs. placebo 16.2%, p = 0.003) (Yang et al., 2022), to primary Sjögren’s syndrome (ESSDAI score Δ = −4.3, p = 0.002) (Xu et al., 2023), and myasthenia gravis (MG) (Guo et al., 2023). Notably, a clinical Phase 2b study published in Journal Name reported that, in patients with SLE, telitacicept demonstrated significant better efficacy than placebo in achieving SRI-4 response at week 48 (71.0%–75.8% vs. 33.9%; all p < 0.001), with comparable safety performance (Wu D. et al., 2024). Furthermore, the Phase III trial in SLE showed 52-week renal response rates of 68.3% while 48.1% with standard therapy (OR 2.32, 95%CI 1.41–3.82) (Cai et al., 2023).

Therefore, we conducted a single-arm retrospective cohort study to extend prior research by evaluating the renoprotective effects of telitacicept in the high-risk lupus nephritis with LN-NS subpopulation, a group underrepresented in pivotal trials.

## Methods

This study recruited patients diagnosed with SLE complicated by LN and NS from 1 January 2020, to 1 November 2023. The patients were sourced from the Departments of Rheumatology and Immunology of the First Affiliated Hospital of Guangzhou University of Chinese Medicine, Foshan Hospital of Traditional Chinese Medicine, and Wuyi Hospital of Traditional Chinese Medicine.

All enrolled patients received weekly subcutaneous injections of telitacicept at a dosage of either 80 mg or 160 mg, with a continuous treatment duration of at least 12 months. Meanwhile, other routine treatments administered, including glucocorticoids, traditional antirheumatic drugs, and non-steroidal anti-inflammatory drugs.

**FIGURE 1:**
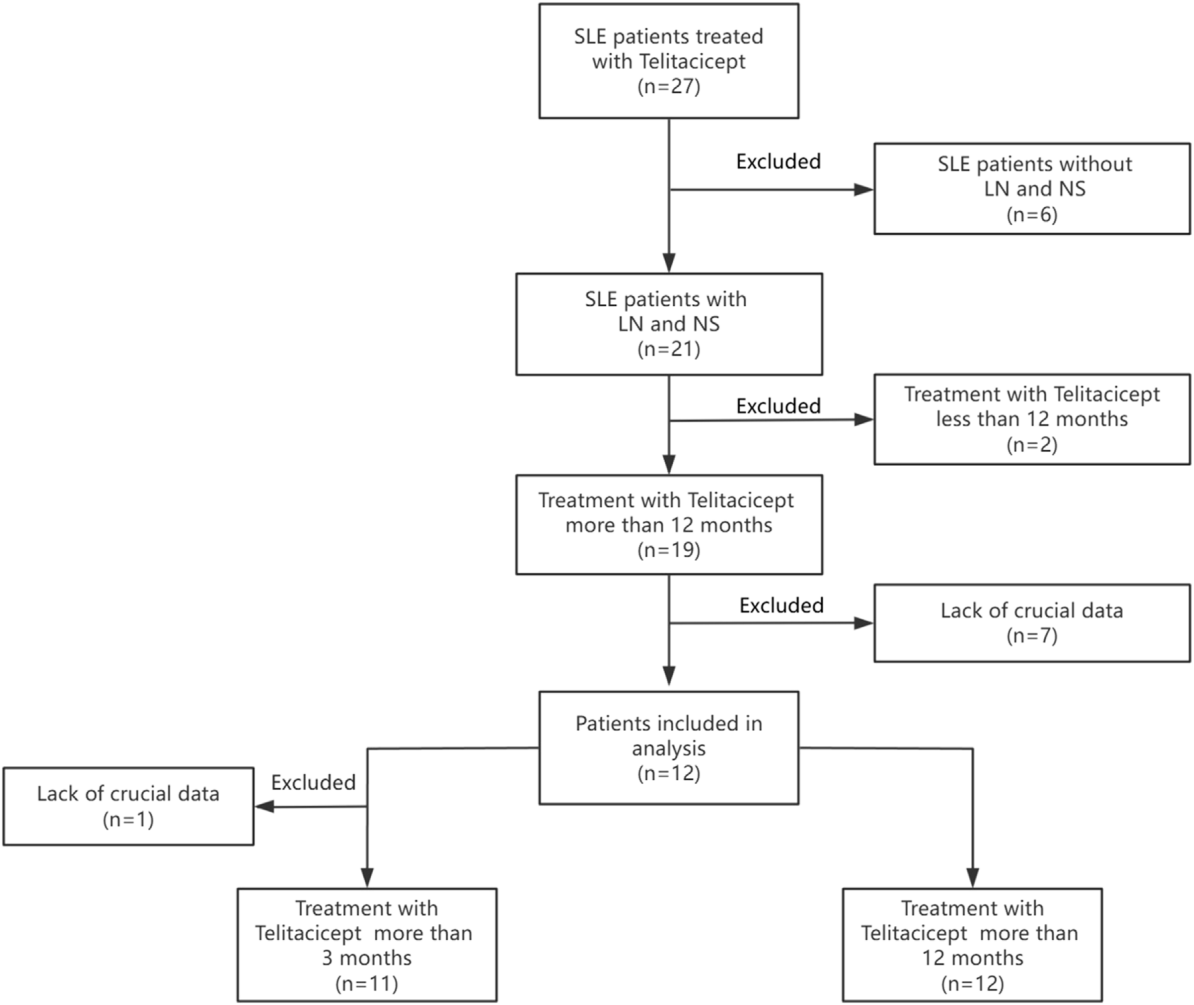
Flowchart of the study design.

In this retrospective analysis, clinical data was systematically collected at standardized intervals of 3 months and 12 months after the initiation of telitacicept treatment. A total of 27 SLE patients treated with telitacicept were initially considered. Patients without LN and NS (n = 6) were excluded, leaving 21 patients with LN and NS. Of these, patients receiving telitacicept for <12 months (n = 2) were excluded, resulting in 19 patients treated for >12 months. Subsequently, patients lacking crucial data (n = 7) were excluded, yielding 12 patients included in the primary analysis. Finally, one additional patient was excluded due to lack of crucial data, resulting in 11 patients treated with telitacicept for >3 months available for further analysis. The study flowchart is presented in Figure 1.

### Study population

We performed a multicenter retrospective cohort study involving patients who met the 2019 American College of Rheumatology/European League Against Rheumatism (ACR/EULAR) classification criteria for SLE. All patients had biopsy-confirmed LN and NS and were treated at three tertiary-care centers, namely, the First Affiliated Hospital of Guangzhou University of Chinese Medicine, Foshan Hospital of Traditional Chinese Medicine, and Jiangmen Traditional Chinese Medicine Hospital, between January 2020 and November 2023.

### Inclusion criteria

1. Patients were required to receive weekly subcutaneous telitacicept administration (either 80 mg or 160 mg) for at least 12 months.
2. Patients had prior renal biopsies with findings consistent with LN.
3. Patients met the diagnostic criteria for NS: 24-h urinary protein >3.5 g/24 h; serum albumin <30 g/L; and edema or hyperlipidemia.
4. Concurrent conventional therapy was required, which comprised immunosuppressants such cyclophosphamide (CsA) or mycophenolate mofetil (MMF), glucocorticoids (prednisone equivalent ≥0.5 mg/kg/day), and angiotensin-converting enzyme inhibitors (ACEi) or angiotensin II receptor blockers (ARBs).
5. Complete 12-month follow-up data were necessary for inclusion in the study.

### Exclusion criteria

1. Patients who experienced adverse events of grade ≥3 according to the Common Terminology Criteria for Adverse Events (CTCAE) version 5.0 were excluded.
2. Patients undergoing concurrent biologics or B-cell depletion therapy were excluded in this study.
3. Pregnant patients or those receiving renal replacement therapy were excluded from the analysis.

### Observational endpoints

This retrospective cohort study evaluated therapeutic responses by analyzing longitudinal changes in key clinical parameters among 12 patients treated with telitacicept. Efficacy assessments were anchored to baseline values obtained ≤72 h prior to treatment initiation.

### Primary endpoint

Composite outcome at 12 months post-treatment including:

• Systemic Lupus Erythematosus Disease Activity Index 2000 (SLEDAI)
• 24-h urinary protein excretion (24hUpr)
• Serum complement components (C3/C4)
• Anti-double stranded DNA (anti-dsDNA) titers
• Immunoglobulin profiles (immunoglobulin G/immunoglobulin M)
• Serum creatinine (SCr)

Secondary Endpoint:

Identical parameters assessed at 3-month interim analysis.

### Safety assessments

Safety assessments included adverse events (AEs), which were meticulously recorded and evaluated using the Common Terminology Criteria for Adverse Events (CTCAE) for grading severity.

### Evaluation of efficacy

SPSS 26.0 software was used for baseline demographics and disease characteristics data analysis. For measurements that conform to a normal distribution, the mean is expressed as ± standard deviation. The non-normal distribution data was presented as the median (IQR). The significance level was p < 0.05. Statistical charts were plotted using GraphPad Prism 8 software.

Effect sizes for each index variable were calculated as the natural logarithm (base e) of the ratio, i.e., $\log\left(\frac{b}{a}\right)$ , where ‘*b’* and ‘*a’* refers to the value before and after the treatment. For SLEDAI index, effect sizes were calculated using the difference before to after the treatment, as this index is a scoring system that evaluates the degree of disease activity in SLE patients. For the C3, C4, and HGB indices, effects were calculated using the reversed log ratio, as these values are expected to increase post-treatment. The significance levels were assessed using a one-sample t-test of the log ratios, with the null hypothesis stating that there is no effect. Analyses were conducted using the R language (R Core Team, 2021).

## Results

In this study, a total of 12 patients were included for analysis. The median age of all participants was 30 (interquartile range [IQR] 24–41), with a median SLE duration of 3 years (IQR 1–8). Body mass index (BMI) was 21.28 (standard deviation [SD] 4.03). Systolic blood pressure was 130.50 mmHg (SD 11.71) and diastolic blood pressure was 77.17 mmHg (SD 9.98). The cohort was predominantly female (91.7%, 11/12), and all participants were of East Asian ethnicity. The majority of patients (83.3%) received a telitacicept dosage of 160 mg per week. Regarding concomitant medications, all patients received corticosteroid therapy. Other concomitant medications included mycophenolate mofetil (MMF) (33.3%), hydroxychloroquine (HCQ) (75.0%), ciclosporin (CYC) (33.3%), methotrexate (MTX) (16.7%), and tacrolimus (TAC) (25.0%) (Table 1) (Supplementary Table S1).

**TABLE 1:** Baseline demographics and disease characteristics of all patients (n = 12).

Classification	Parameters	All (n = 12)
Basic information	Sex, n (%, female)	91.7
	Age (years)	30 (24, 41)
	SLE Duration (years)	3 (1, 8)
	Dosage, n (%, 160 mg/qw)	83.3
	BMI	21.28 (4.03)
	Systolic blood pressure (mm/Hg)	130.50 (11.71)
	Diastolic blood pressure (mm/Hg)	77.17 (9.98)
Clinical indicators	WBC (10^9^/L)	7.32 (4.66, 8.43)
	NEU (10^9^/L)	4.59 (2.82, 5.10)
	LYM (10^9^/L)	1.73 (1.01, 2.80)
	RBC (10^12^/L)	4.29 (1.08)
	HGB (g/L)	107 (19.97)
	PLT (10^9^/L)	250 (212, 371)
	AST (U/L)	19 (13, 34)
	ALT (U/L)	23 (7, 32)
	Urea (mmol/L)	5.21 (3.64, 8.33)
	Glu (mmol/L)	4.87 (0.90)
	UA (μmol/L)	360 (79.39)
Concomitant medications	Glucocorticoids (%)	100
	MMF (%)	33.3
	HCQ (%)	75.0
	CYC (%)	33.3
	MTX (%)	16.7
	TAC (%)	25.0

NEU, neutrophilic granulocyte; LYM, lymphocyte; RBC, erythrocytes; HGB, hemoglobin; PLT, platelet; UA, uric acid; MMF, mycophenolate mofetil; HCQ, hydroxychloroquine; CYC, ciclosporin; MTX, methotrexate; TAC, tacrolimus. Continuous variables with normal distribution are expressed as mean ± SD, and non-normally distributed variables are summarized as median (IQR).

Prior to telitacicept initiation, the 12 patients’ baseline clinical indicators were as follows: white blood cell count (WBC) 7.32 (IQR 4.66–8.43) ×10^9^/L, hemoglobin (HGB) 107 g/L (SD 19.97), platelet count (PLT) 250 (IQR 212–371) ×10^9^/L, aspartate aminotransferase (AST) 19 U/L (IQR 13–34), alanine aminotransferase (ALT) 23 U/L (IQR 7–32), serum urea 5.21 mmol/L (IQR 3.64–8.33), fasting blood glucose (Glu) 4.87 mmol/L (SD 0.90), and serum uric acid (UA) 360 μmol/L (SD 79.39). These baseline parameters indicated that WBC, PLT, liver function (AST, ALT), fasting blood glucose, serum uric acid, and serum urea levels were within normal ranges for all 12 patients, with the exception of hemoglobin, which was below the normal reference range. (Table 1).

**FIGURE 2:**
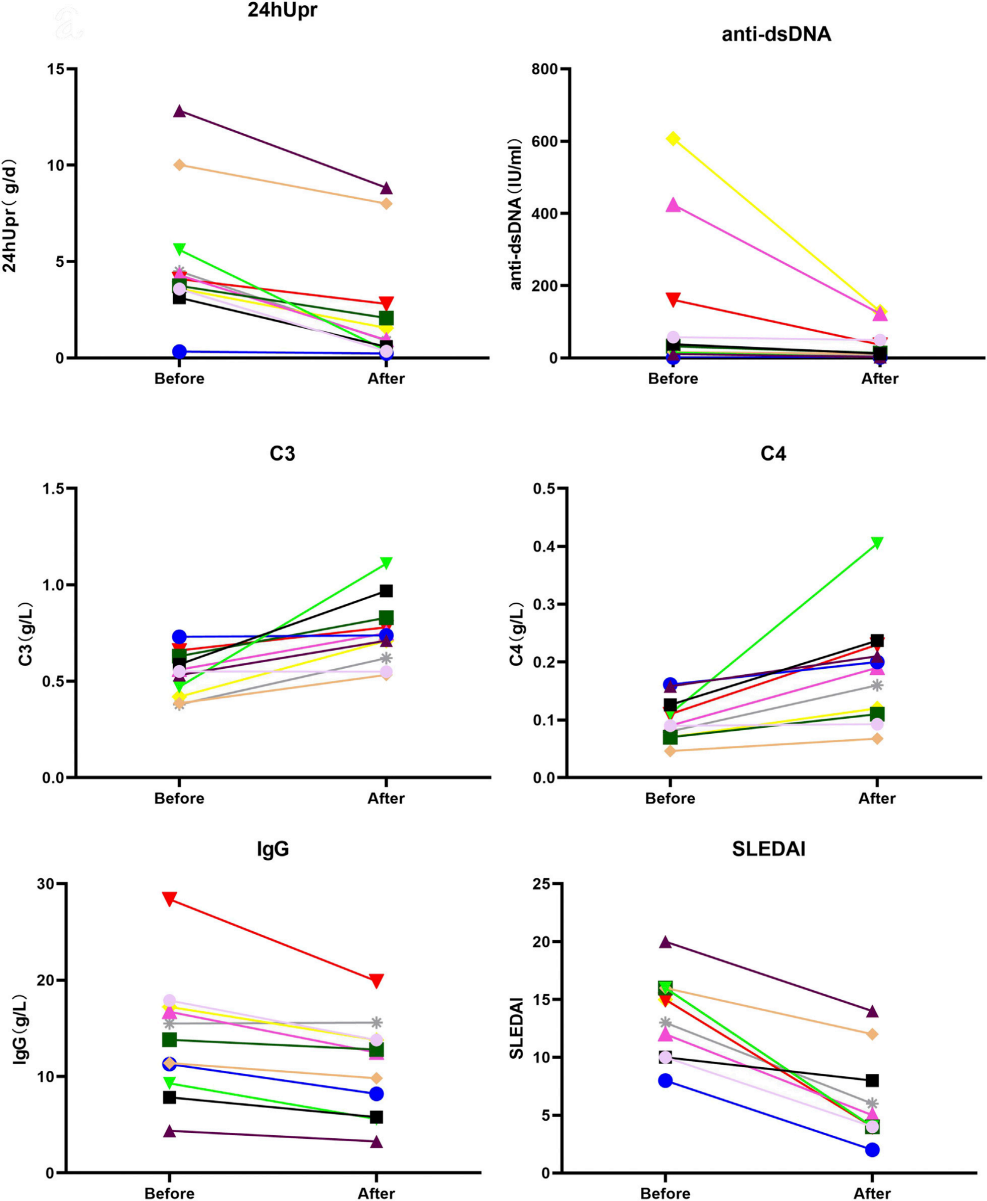
Comparison of SLEDAI, 24hUpr, C3, C4, IgG and anti-dsDNA before and after 3-month treatment with telitacicept.

**FIGURE 3:**
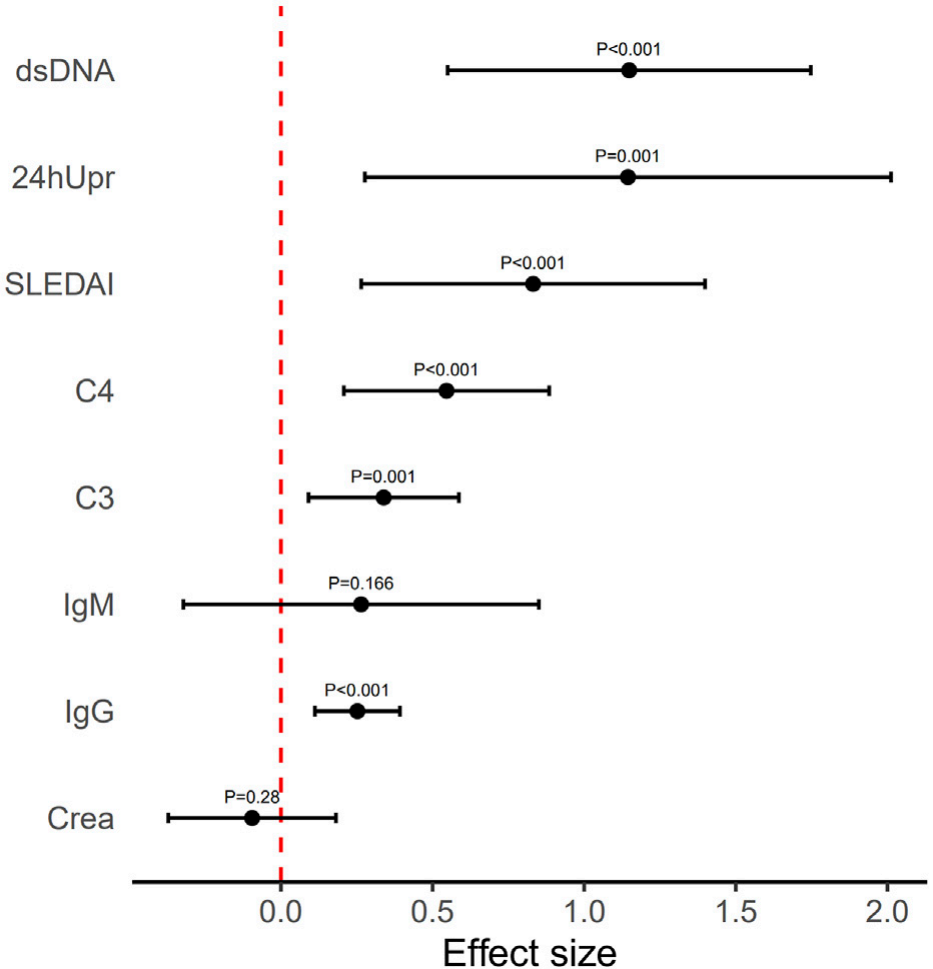
Mean effect size observed for each variable index following 3-month telitacicept treatment. Error bars refer to mean and standard deviation.

After 3 months of telitacicept therapy, changes in key disease activity and serological parameters, including the SLEDAI, 24hUpr, C3 and C4, anti-dsDNA, IgG, IgM, and SCr, were compared pre- and post-treatment (Figure 2). Effect sizes were calculated to quantify treatment efficacy across these clinical variables. As demonstrated in Figure 3 and Table 2, telitacicept significantly reduced SLEDAI scores, 24hUP, anti-dsDNA levels, and IgG concentrations, while markedly increasing C3 and C4 levels. In contrast, IgM and SCr showed no big changes after treatment. Notably, important reductions from baseline were observed for anti-dsDNA, SLEDAI, C4 and IgG levels.

**TABLE 2:** The mathematical mean values and their range (minimum and maximum values) were analyzed across all patients for each index before and after the 3-month treatment. The effect size represents the mean and SD of the log ratio (i.e., equivalent to the geometric mean of the original values), calculated from before to after the treatment. For indices C3 and C4 (indicated by *), effect sizes were calculated using the log ratio from after to before the treatment. P-values were derived from a t-test comparing the effect sizes to zero.

Index	Before (mean and range)	After (mean and range)	Effect (mean and SD)	p
dsDNA	140 (11–610)	35 (1–130)	1.15 (0.6)	<0.001
24hUpr	5.1 (0.33–13)	2.4 (0.24–8.8)	1.14 (0.87)	0.001
SLEDAI	12 (8–16)	6.1 (2–14)	0.83 (0.57)	<0.001
C4	0.1 (0.046–0.16)	0.18 (0.068–0.4)	0.55 (0.34)*	<0.001
C3	0.54 (0.38–0.73)	0.75 (0.53–1.1)	0.34 (0.25)*	0.001
IgM	2 (0.72–6.2)	1.8 (0.26–5.6)	0.26 (0.59)	0.166
IgG	14 (4.4–28)	11 (3.3–20)	0.25 (0.14)	<0.001
Crea	68 (41–170)	73 (45–150)	−0.1 (0.28)	0.280

**FIGURE 4:**
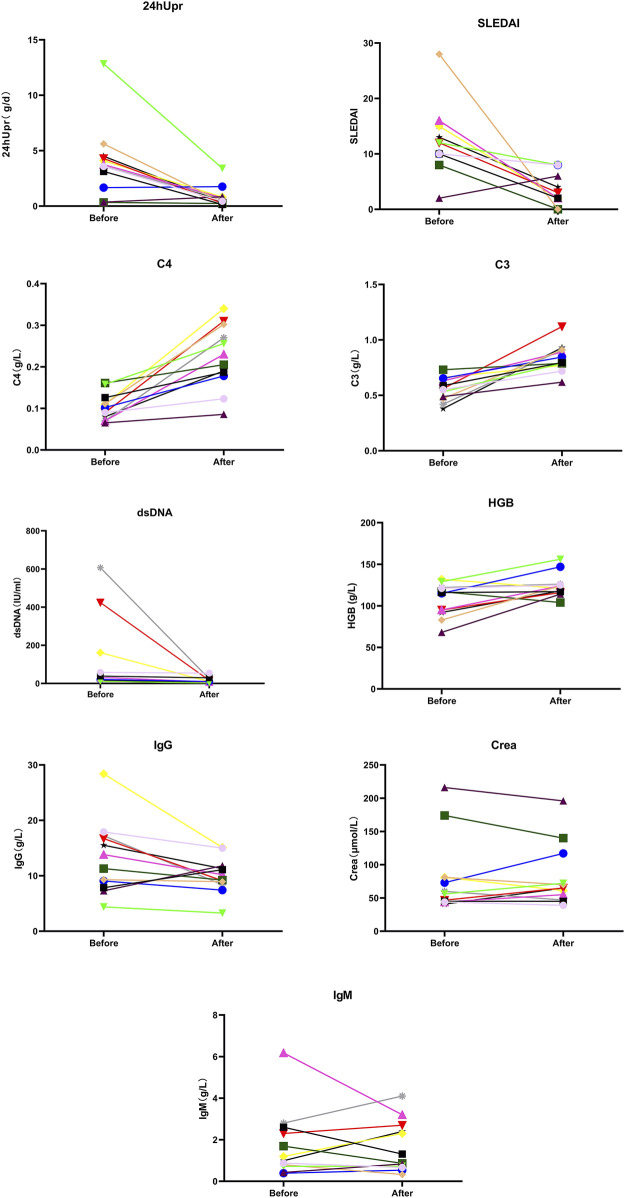
Comparison of SLEDAI, 24 hUpr, C3, C4, dsDNA, IgG, IgM, Crea and HGB before and after 12-month treatment with telitacicept.

**FIGURE 5:**
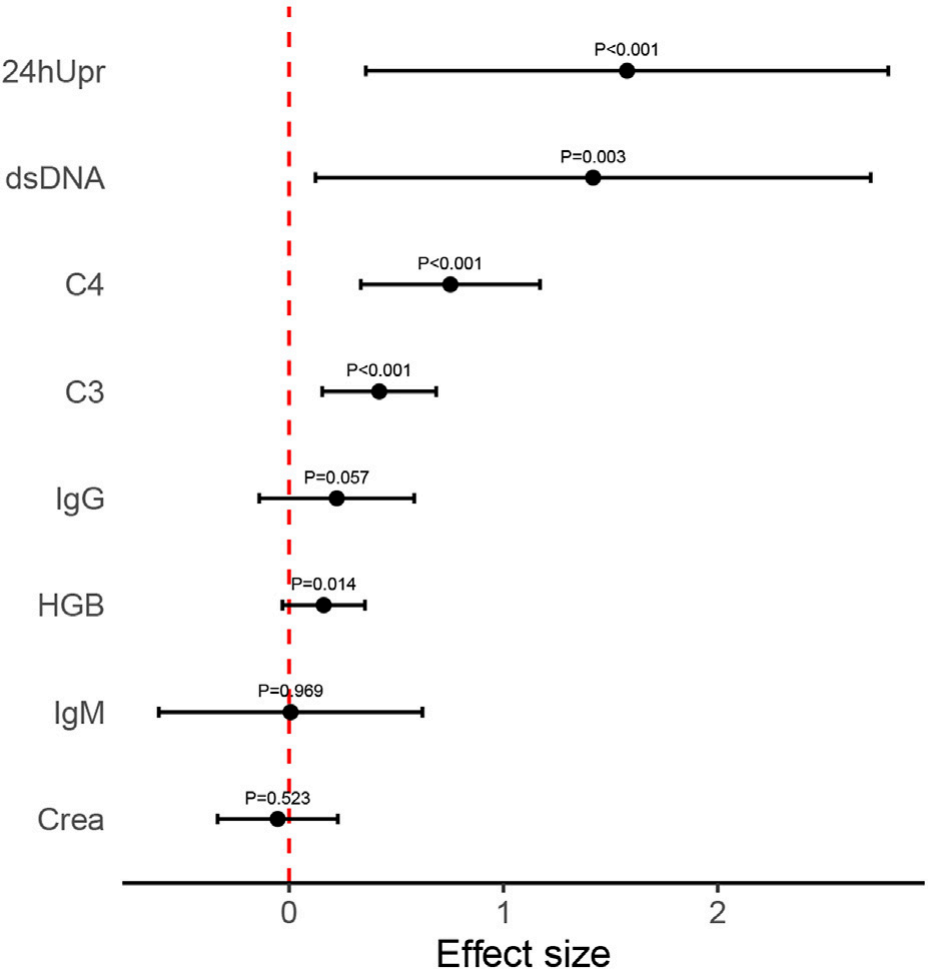
Mean effect size observed for each variable index following 12-month telitacicept treatment. Error bars refer to mean and standard deviation.

After 12 months of telitacicept treatment, significant changes were observed not only in key markers (SLEDAI, 24hUpr, C3, C4, dsDNA, IgG, IgM, SCr) but also in HGB (Figure 4). Effect sizes were calculated to quantify treatment efficacy across clinical variables. As shown in Figure 5 and Table 3, telitacicept considerably reduced SLEDAI, 24hUpr, and dsDNA levels while increasing C3, C4, and HGB. In contrast, IgM, IgG, and SCr remained stable post-treatment. Notably, 24hUpr demonstrated a significant decrease from baseline, whereas C3 and C4 showed important increases (Figure 5; Table 3).

**TABLE 3:** The mathematical mean values and their range (minimum and maximum values) across all patients for each index before and after the 12-month treatment. The effect size represents the mean and SD of the log ratio (i.e., equivalent to the geometric mean of the original values), calculated from before to after the treatment. For SLEDAI index, effect sizes were calculated using the difference before to after the treatment (indicated by the superscript letter ‘a’); for C3, C4 and HGB, effect sizes were calculated using the log ratio from after to before the treatment (indicated by the superscript letter ‘b’). P-values were derived from a t-test comparing the effect sizes to zero.

Index	Before (mean and range)	After (mean and range)	Effect (mean and SD)	p
SLEDAI	13 (2–28)	3.8 (0–8)	8.83 (8.04)^a^	0.003
24hUpr	4 (0.33–13)	0.83 (0.15–3.4)	1.58 (1.22)	<0.001
dsDNA	120 (1–610)	13 (1–54)	1.42 (1.3)	0.003
C4	0.1 (0.065–0.16)	0.22 (0.086–0.34)	0.75 (0.42)^b^	<0.001
C3	0.56 (0.38–0.73)	0.84 (0.62–1.1)	0.42 (0.27)^b^	<0.001
IgG	13 (4.4–28)	10 (3.3–15)	0.22 (0.36)	0.057
IgM	1.7 (0.39–6.2)	1.7 (0.33–4.1)	0.01 (0.62)	0.100
Crea	80 (41–220)	81 (39–200)	−0.05 (0.28)	0.523
HGB	110 (68–130)	120 (100–160)	0.16 (0.19)^b^	0.014

^a^ effect sizes were calculated using the difference before to after the treatment.

^b^ effect sizes were calculated using the log ratio from after to before the treatment.

## Discussion

SLE is a prototypical autoimmune disorder. It is distinguished by the involvement of multiple systemic organs and the presence of various antibodies in the patient’s bloodstream. Owing to the pronounced heterogeneity inherent of the disease and the substantial clinical disparities among SLE patients, achieving consistent therapeutic outcomes remains challenging. (Arbitman et al., 2022).

LN is a prevalent complication of SLE. It can manifest asymptomatically or present with proteinuria, hematuria, and impaired renal function. LN continues to be a significant risk factor for chronic kidney injury and end-stage renal disease in SLE patients, contributing to considerable morbidity and mortality (Steiger et al., 2022; Chang et al., 2021).

As a crucial component of the human immune system, B cells mainly generate immune responses through antibody secretion. They also interacts with T cells to secrete cytokines, which play an important role in the initiation and progression of autoimmunity. Throughout the pathogenesis of SLE, abnormal B-cell over-activation is a characteristic feature (Kang et al., 2022; Yap and Chan, 2019). Once activated, B cells differentiate into plasma cells, also referred to as antibody-secreting cells or effector B cells (Wu et al., 2023). Plasma cells are highly active and produce an excessive amount of autoantibodies. These antibodies are essential in driving immune complex formation and deposition, which in turn provoke inflammation and cause tissue damage.

Both BAFF and April belong to the tumor necrosis factor (TNF) family of cytokines, which are critical for B-cell growth and proliferation (Vincent et al., 2013). Therapies targeting abnormal B-cell activation have become a popular strategy in the clinical management of SLE (Bag-Ozbek and Hui-Yuen, 2021).

Belimumab is a recombinant, soluble humanized monoclonal antibody against BAFF. It blocks the binding of BAFF to its receptors, inhibits B-cell activation, and reduces antibody production. Since 2011, the U.S. Food and Drug Administration (FDA) has approved belimumab for SLE patients with active, autoantibody-positive disease. In several studies, belimumab demonstrated efficacy in patients with LN (Plüß et al., 2022; Sciascia et al., 2017). Related research indicated that, compared with the control group, belimumab can alleviate symptoms and improve renal function in LN patients (Furie et al., 2020); however, its onset of action seems to be delayed (Zhang et al., 2023). Other studies suggested that belimumab can help mitigate arthritis, rash, and thrombocytopenia. Additionally, belimumab treatment also reduces the likelihood of serious treatment-related adverse events and the need for high-dose glucocorticoids (Wu Q. et al., 2024; Chan et al., 2023; Zen et al., 2023).

Telitacicept, a dual-target biological agent, can simultaneously bind to both April and BAFF, effectively impeding their interaction with respective receptors. Previous research has indicated that in patients with SLE, the serum level of April is positively correlated with the elevation of autoantibodies and the extent of organ damage (Salazar-Camarena et al., 2020). In animal experiments, knocking out the April gene in SLE mice could ameliorate LN and reduce mortality (Tran et al., 2017).

A phase III, placebo-controlled, multi-center, randomized, double-blind study conducted in China to assess the safety and efficacy of telitacicept in comparison to placebo demonstrated that the telitacicept 160 mg group achieved a Systemic Lupus Erythematosus Responder Index 4 (SRI-4) response rate of 82.6% after 52 weeks of treatment. Moreover, all SRI-5/6/7/8 response rates were higher than those in the placebo group.

In this retrospective cohort study, we evaluated the efficacy of telitacicept in patients with SLE complicated by LN and NS over treatment durations of 3 months and 12 months, in addition to conventional therapy.

After 3 months of telitacicept treatment, a significant reduction in the SLEDAI, anti-dsDNA levels, and IgG levels were observed. There was also a decrease in 24hUpr and an increase in complement C3 and complement C4. Notably, patients with higher baseline anti-dsDNA levels exhibited a more pronounced decline after 3 months of telitacicept treatment. No significant differences were found in SCr and IgM levels during treatment. That the possible explanation is that, in most patients, SCr levels were not severely elevated and IgM levels were within the normal range before treatment. The reductions in anti-dsDNA, SLEDAI scores, and IgG levels, coupled with the increases in complement components C3 and C4, demonstrate a decrease in disease activity for both SLE and LN. Furthermore, the reduction in 24hUpr indicated effective control of LN and associated NS, reflecting an attenuation of renal injury.

After 12 months of telitacicept treatment, a significant decrease of SLEDAI and anti-dsDNA levels were found. Similar to the 3-month results, 24hUpr decreased, and C3 and C4 increased. However, a significant increase in HGB levels was noted, while IgG levels remained relatively stable. Twelve patients showed a statistically significant improvement in HGB levels after 12 months of telitacicept treatment, which is consistent with previous reports (Cheng et al., 2024; Jin et al., 2025). In particular, 5 patients with moderate anemia (baseline HGB levels ranging from 60 to 90 g/L) exhibited a substantial elevation in HGB, with all reaching normal levels within the reference range. Although the evidence on telitacicept’s role in ameliorating anemia remains limited, current data strongly indicate its potential effectiveness. These findings highlight the necessity for further mechanistic and clinical investigations to clarify the potential pathways through which telitacicept confers hematologic benefits and confirm its therapeutic value in the management of anemia.

Although no statistically significant changes in IgG levels were observed in the 12 months, a clinically meaningful trend emerged. Before treatment, only four patients had elevated IgG levels (16.73–28.39 g/L). After telitacicept administration, all patients’ IgG concentrations fell within the normal reference range (IgG <15.6 g/L), implying a potential regulatory effect of the therapy on immunoglobulin homeostasis, despite the lack of statistical significance. Moreover, IgG reduction was significant at 3 months post-telitacicept initiation, indicating a rapid treatment effect on IgG suppression within the initial treatment phase.

This study demonstrated the significant efficacy of telitacicept in reducing proteinuria, enhancing renal function, alleviating the activity of SLE, and improving the anemic state of patients. In terms of safety, clinical follow-up and auxiliary examinations showed that during the entire observation period, none of the 12 enrolled patients experienced treatment-related adverse events, such as infections or gastrointestinal symptoms.

However, this study has several limitations. Firstly, it was a single-arm retrospective study. The small sample size, absence of a control group, and relatively simplistic statistical methods restricted the scope of the conclusions drawn. Given that SLE is a disease with a long treatment course and a high tendency to recur, the short observation period in this study made it impossible to accurately evaluate the long-term efficacy of telitacicept.

Secondly, this study aimed to assess the efficacy of telitacicept in patients with SLE complicated by LN and NS. Nevertheless, the lack of sufficient longitudinal follow-up data prevented a comparative analysis of serum albumin levels, blood pressure, and lipid profiles before and after treatment.

In summary, future research should carefully reconsider the study design, conduct more comprehensive patient follow-up, increase the sample size, and carry out further in-depth investigations.

In conclusion, this rpractical cohort study provides new evidence for the treatment of renal involvement in SLE. Telitacicept demonstrated clinical efficacy and a favorable safety profile in the treatment of SLE with LN and NS in this cohort.

## Data Availability

The raw data supporting the conclusions of this article will be made available by the authors, without undue reservation.

## References

[B1] Arbitman L. Furie R. Vashistha H. (2022). B cell-targeted therapies in systemic lupus erythematosus. J. Autoimmun. 132, 102873. 10.1016/j.jaut.2022.102873 35963808

[B2] Bag-Ozbek A. Hui-Yuen J. S. (2021). Emerging B-Cell therapies in systemic lupus erythematosus. Ther. Clin. Risk Manag. 17, 39–54. 10.2147/TCRM.S252592 33488082 PMC7814238

[B3] Basta F. Fasola F. Triantafyllias K. Schwarting A. (2020). Systemic lupus erythematosus (SLE) therapy: the old and the new. Rheumatol. Ther. 7 (3), 433–446. 10.1007/s40744-020-00212-9 32488652 PMC7410873

[B4] Cai J. Gao D. Liu D. Liu Z. (2023). Telitacicept for autoimmune nephropathy. Front. Immunol. 14, 1169084. 10.3389/fimmu.2023.1169084 37342346 PMC10277628

[B5] Chan J. Walters G. D. Puri P. Jiang S. H. (2023). Safety and efficacy of biological agents in the treatment of systemic lupus erythematosus (SLE). BMC Rheumatol. 7 (1), 37. 10.1186/s41927-023-00358-3 37807057 PMC10561476

[B6] Chang A. Clark M. R. Ko K. (2021). Cellular aspects of the pathogenesis of lupus nephritis. Curr. Opin. Rheumatology 33 (2), 197–204. 10.1097/BOR.0000000000000777 PMC7905798 33394604

[B7] Chen Y. Shi N. Lei X. Ren P. Lan L. Chen L. (2023). The efficacy of rituximab plus belimumab or telitacicept in refractory lupus nephritis. Rheumatol. Oxf. 64, 221–227. 10.1093/rheumatology/kead674 38145455

[B8] Cheng J. Peng Y. Wu Q. Wu Q. He J. Yuan G. (2024). Efficacy and safety of telitacicept therapy in systemic lupus erythematosus with hematological involvement. Clin. Rheumatol. 43 (7), 2229–2236. 10.1007/s10067-024-06992-7 38767710

[B9] Dhillon S. (2021). Telitacicept: first approval. Drugs 81 (14), 1671–1675. 10.1007/s40265-021-01591-1 34463932

[B10] Fava A. Petri M. (2019). Systemic lupus erythematosus: diagnosis and clinical management. J. Autoimmun. 96, 1–13. 10.1016/j.jaut.2018.11.001 30448290 PMC6310637

[B11] Frățilă V. G. Lupușoru G. Sorohan B. M. Obrișcă B. Mocanu V. Lupușoru M. (2024). Nephrotic syndrome: from pathophysiology to novel therapeutic approaches. Biomedicines 12 (3), 569. 10.3390/biomedicines12030569 38540182 PMC10968602

[B12] Furie R. Rovin B. H. Houssiau F. Malvar A. Teng Y. K. O. Contreras G. (2020). Two-year, randomized, controlled trial of belimumab in lupus nephritis. N. Engl. J. Med. 383 (12), 1117–1128. 10.1056/NEJMoa2001180 32937045

[B13] Gasparotto M. Gatto M. Binda V. Doria A. Moroni G. (2020). Lupus nephritis: clinical presentations and outcomes in the 21st century. Rheumatology 59 (Suppl. ment_5), v39–v51. 10.1093/rheumatology/keaa381 33280015 PMC7751166

[B14] Guo Q. Huang Y. Wang F. Fang L. (2023). Case report: telitacicept in severe myasthenia gravis: a case study with multiple autoantibodies. Front. Immunol. 14, 1270011. 10.3389/fimmu.2023.1270011 38124751 PMC10731252

[B15] Jin H. Z. Cai M. L. Wang X. Li Z. Niu L. Wang P. (2025). Effectiveness and safety of belimumab and telitacicept in systemic lupus erythematosus: a real-world, retrospective, observational study. Clin. Rheumatol. 44 (1), 247–256. 10.1007/s10067-024-07266-y 39680262

[B16] Kang N. Liu X. You X. Sun W. Haneef K. Sun X. (2022). Aberrant B-Cell activation in systemic lupus erythematosus. Kidney Dis. (Basel) 8 (6), 437–445. 10.1159/000527213 36590680 PMC9798842

[B17] Parra Sánchez A. R. Voskuyl A. E. van Vollenhoven R. F. (2022). Treat-to-target in systemic lupus erythematosus: advancing towards its implementation. Nat. Rev. Rheumatol. 18 (3), 146–157. 10.1038/s41584-021-00739-3 35039665

[B18] Plüß M. Piantoni S. Tampe B. Kim A. H. J. Korsten P. (2022). Belimumab for systemic lupus erythematosus-Focus on lupus nephritis. Hum. Vaccin Immunother. 18 (5), 2072143. 10.1080/21645515.2022.2072143 35588699 PMC9359396

[B19] R Core Team (2021). R: a language and environment for statistical computing. Vienna, Austria: R Foundation for Statistical Computing.

[B20] Salazar-Camarena D. C. Palafox-Sánchez C. A. Cruz A. Marín-Rosales M. Muñoz-Valle J. F. (2020). Analysis of the receptor BCMA as a biomarker in systemic lupus erythematosus patients. Sci. Rep. 10 (1), 6236. 10.1038/s41598-020-63390-0 32277232 PMC7148319

[B21] Sciascia S. Radin M. Yazdany J. Levy R. A. Roccatello D. Dall'Era M. (2017). Efficacy of belimumab on renal outcomes in patients with systemic lupus erythematosus: a systematic review. Autoimmun. Rev. 16 (3), 287–293. 10.1016/j.autrev.2017.01.010 28147262

[B22] Shi F. Xue R. Zhou X. Shen P. Wang S. Yang Y. (2021). Telitacicept as a BLyS/APRIL dual inhibitor for autoimmune disease. Immunopharmacol. Immunotoxicol. 43 (6), 666–673. 10.1080/08923973.2021.1973493 34519594

[B23] Steiger S. Ehreiser L. Anders J. Anders H. J. (2022). Biological drugs for systemic lupus erythematosus or active lupus nephritis and rates of infectious complications. Evidence from large clinical trials. Front. Immunol. 13, 999704. 10.3389/fimmu.2022.999704 36211360 PMC9538665

[B24] Tian J. Zhang D. Yao X. Huang Y. Lu Q. (2023). Global epidemiology of systemic lupus erythematosus: a comprehensive systematic analysis and modelling study. Ann. rheumatic Dis. 82 (3), 351–356. 10.1136/ard-2022-223035 PMC9933169 36241363

[B25] Tran N. L. Schneider P. Santiago-Raber M. L. (2017). TACI-Dependent APRIL signaling maintains autoreactive B cells in a mouse model of systemic lupus erythematosus. Eur. J. Immunol. 47 (4), 713–723. 10.1002/eji.201646630 28267197

[B26] Vincent F. B. Saulep-Easton D. Figgett W. A. Fairfax K. A. Mackay F. (2013). The BAFF/APRIL system: emerging functions beyond B cell biology and autoimmunity. Cytokine and Growth Factor Rev. 24 (3), 203–215. 10.1016/j.cytogfr.2013.04.003 23684423 PMC7108297

[B27] Wu D. Li J. Xu D. Merrill J. T. van Vollenhoven R. F. Liu Y. (2024). Telitacicept in patients with active systemic lupus erythematosus: results of a phase 2b, randomised, double-blind, placebo-controlled trial. Ann. rheumatic Dis. 83, 4 475–487. 10.1136/ard-2023-224854 PMC10958275 38129117

[B28] Wu L. Du X. Lu X. (2023). Role of telitacicept in the treatment of IgA nephropathy. Eur. J. Med. Res. 28 (1), 369. 10.1186/s40001-023-01320-2 37737205 PMC10515419

[B29] Wu Q. Zhao M. X. Huang X. S. Lin C. S. Xu Q. (2024). The use of belimumab on patients with both systemic lupus erythematosus and immune thrombocytopenia: a retrospective cohort study. Lupus 33 (6), 608–614. 10.1177/09612033241241576 38518059

[B30] Xu D. Fang J. Zhang S. Huang C. Huang C. Qin L. (2023). Efficacy and safety of telitacicept in primary Sjögren’s syndrome: a randomized, double-blind, placebo-controlled, phase 2 trial. Rheumatology 63, 698–705. 10.1093/rheumatology/kead265 37399108

[B31] Yang J. Gao J. Zhao W. J. Xu Q. Li H. Y. Yu C. (2022). Research progress of telitacicept in IgA nephrophy and lupus nephritis. China J. Mod. Med. 32 (19), 51–56. (Chinese with English Abstract).

[B32] Yap D. Y. H. Chan T. M. (2019). B cell abnormalities in systemic lupus erythematosus and lupus nephritis—role in pathogenesis and effect of immunosuppressive treatments. Int. J. Mol. Sci. 20, 6231. 10.3390/ijms20246231 31835612 PMC6940927

[B33] Zen M. Gatto M. Depascale R. Regola F. Fredi M. Andreoli L. (2023). Early and late response and glucocorticoid-sparing effect of belimumab in patients with systemic lupus erythematosus with joint and skin manifestations: results from the belimumab in real life setting study-joint and skin (BeRLiSS-JS). J. Pers. Med. 13 (4), 691. 10.3390/jpm13040691 37109077 PMC10146447

[B34] Zhang K. Qi T. Guo D. Liu Y. (2023). Efficacy and safety of belimumab therapy for patients with lupus nephritis: a meta-analysis and a propensity score-matched case-control study. Immun. Inflamm. Dis. 11 (7), e954. 10.1002/iid3.954 37506137 PMC10373564

[B35] Zucchi D. Silvagni E. Elefante E. Signorini V. Cardelli C. Trentin F. (2023). Systemic lupus erythematosus: one year in review 2023. Clin. Exp. Rheumatol. 41 (5), 997–1008. 10.55563/clinexprheumatol/4uc7e8 37133502

