# Supplementary material for: Efficacy and safety of telitacicept in systemic lupus erythematosus with lupus nephritis and nephrotic syndrome: a 12-month retrospective cohort study
Source: Front Pharmacol. 2025 Jul 18;16:1613790. doi: 10.3389/fphar.2025.1613790 (PMC12313615; doi:10.3389/fphar.2025.1613790)
Supplement: Supplementary file 1 [file Table1.docx]

Supplement Table 1 Basic information, comorbidities and concomitant medications of the 12 patients.

| NO. | Sex | Age  (years) | SLE Duration  (years) | Dosage,(mg/qw) | BMI | Blood pressure(mm/Hg) | Concomitant medications |
| --- | --- | --- | --- | --- | --- | --- | --- |
| 1 | Female | 34 | 9 | 80 | 22 | 109/73 | Glucocorticoids, TAC |
| 2 | Female | 25 | 6 | 80 | 17.2 | 130/77 | Glucocorticoids, TAC, HCQ |
| 3 | Female | 23 | 3 | 160 | 15 | 129/70 | Glucocorticoids, CYC, HCQ |
| 4 | Female | 20 | 8 | 160 | 30.2 | 137/92 | Glucocorticoids, CYC, TAC |
| 5 | Female | 49 | 1 | 160 | 23 | 154/91 | Glucocorticoids, HCQ, MMF |
| 6 | Female | 36 | 13 | 160 | 18.1 | 146/93 | Glucocorticoids, MMF, HCQ |
| 7 | Female | 26 | 2 | 160 | 21.6 | 133/77 | Glucocorticoids, MMF, HCQ |
| 8 | Female | 32 | 0.3 | 160 | 23.3 | 128/79 | Glucocorticoids, HCQ, CYC |
| 9 | Female | 20 | 2 | 160 | 20.2 | 126/68 | Glucocorticoids, MTX |
| 10 | Male | 61 | 6 | 160 | 25.4 | 130/75 | Glucocorticoids, MTX, HCQ |
| 11 | Female | 27 | 3 | 160 | 18.7 | 118/65 | Glucocorticoids, HCQ, MMF |
| 12 | Female | 42 | 1 | 160 | 20.6 | 126/66 | Glucocorticoids, CYC, HCQ |
