# Supplementary material for: Efficacy and safety of telitacicept in systemic lupus erythematosus with lupus nephritis and nephrotic syndrome: a 12-month retrospective cohort study
Source: Front Pharmacol. 2025 Jul 18;16:1613790. doi: 10.3389/fphar.2025.1613790 (PMC12313615; doi:10.3389/fphar.2025.1613790)
Supplement: Supplementary file 2 [file Supplementaryfile1.doc]

STROBE Statement—checklist of items that should be included in reports of observational studies

|  | Item No | Performance |
| --- | --- | --- |
| **Title and abstract** | 1 | √ |
| Introduction |  | |
| Background/rationale | 2 | √ |
| Objectives | 3 | √ |
| Methods |  | |
| Study design | 4 | √ |
| Setting | 5 | √ |
| Participants | 6 | √ |
| Variables | 7 | √ |
| Data sources/ measurement | 8 | √ |
| Bias | 9 | √ |
| Study size | 10 | √ |
| Quantitative variables | 11 | √ |
| Statistical methods | 12 | √ |
| Results |  |  |
| Participants | 13 | √ |
| Descriptive data | 14 | √ |
| Outcome data | 15 | √ |
| Main results | 16 | √ |
| Other analyses | 17 | √ |
| Discussion |  |  |
| Key results | 18 | √ |
| Limitations | 19 | √ |
| Interpretation | 20 | √ |
| Generalisability | 21 | √ |
| Other information |  |  |
| Funding | 22 | √ |
